# Trends in the Prevalence of Atrial Septal Defect and Its Associated Factors among Congenital Heart Disease Patients in Vietnam

**DOI:** 10.3390/jcdd7010002

**Published:** 2019-12-27

**Authors:** Ho Xuan Tuan, Phan The Phuoc Long, Vu Duy Kien, Le Manh Cuong, Nguyen Van Son, Robert Dalla-Pozza

**Affiliations:** 1 Center for International Health, Ludwig-Maximilians-Universität München, 80539 Munich, Germany; robertdallapozza@gmail.com; 2 School of Medicine and Pharmacy, Da Nang University, Da Nang 550000, Vietnam; dr.long67@gmail.com; 3 OnCare Medical Technology Company Limited, Hanoi 100000, Vietnam; vuduykien@gmail.com; 4 National Hospital of Traditional Medicine, Hanoi 100000, Vietnam; drcuong68@gmail.com; 5 Phu Tho Provincial General Hospital, Viet Tri 290000, Vietnam; nguyensonbs@gmail.com

**Keywords:** congenital heart disease, atrial septal defect, prevalence, trends, Vietnam

## Abstract

Atrial septal defect (ASD) is a non-physiologic communication between the two atria, allowing the shunt between systemic and pulmonary circulation. Data about ASD prevalence among congenital heart disease patients (CHD) in Vietnam are still scarce. We aim to assess the trends in the prevalence of ASD patients and associated factors among CHD patients. This was a cross-sectional study, with data collected from medical records from 1220 CHD patients in Da Nang hospital from 1 January 2010 to 31 December 2015. Descriptive statistics were used to estimate the prevalence of ASD among CHD patients. Comparative statistical methods were used to compare groups and logistic regression to access associated factors with ASD. The overall prevalence of ASD among CHD patients was 18.5% between 2010 and 2015. The prevalence varied between periods, ranging between 15% and 31.9% during the period. The prevalence of ASD women among CHD (25.9%) was significantly higher than for men (16.0%). The prevalence of ASD increased gradually when the age group increased. The factors associated with increased ASD prevalence were being a female and being in an older age group. The findings suggest that targeted policy should provide more-specific health-care services of ASD for women and older patients.

## 1. Introduction

Atrial septal defect (ASD) is a non-physiologic communication between the two atria of the heart, which allows shunting between the systemic and the pulmonary circulations. ASD is among the most common types of congenital heart disease (CHD) [1,2,3,4], being the second most frequent type of CHD (0.07–0.2%) in children [5]. The estimated incidence worldwide is 56 per 100,000 live births and the prevalence of 1.6 per 1000 live births [6,7]. With improved detection of clinically silent defects by echocardiography, the estimated incidence is expected to be of 100 per 100,000 live births [6]. ASD is the most common form of CHD that goes under detected during childhood. In adults, it accounts for 7% to 10% of all CHD and between 20% and 40% of all newly diagnosed CHD [1,8].

In developed countries, surgical and catheter-based interventions in ASD patients have facilitated survival to adulthood and the actual life expectancy is between 50 and 60 years [9]. In the USA, the number of adults with CHD is higher than for children and constitutes 60% of the total CHD population [10]. Therefore, developed Western countries are making additional efforts to properly treat a rapidly growing population of adolescents and adults with ASD [10,11]. In developing countries with economic constraints, health services are less developed, delaying the diagnosis and treatment of patients with ASD. Moreover, data are limited on the characteristics of ASD in countries with the lowest incomes, leading to underestimation of ASD burden and treatment cost for ASD in those countries [12,13].

In Vietnam, some studies on ASD were conducted, but their sample size was small, and they focused more on surgery techniques [14,15,16]. In addition, with the advance and increasing availability of echocardiography, the prevalence of ASD might increase due to improved diagnosis, especially in neonates [17]. However, the data about the status of ASD as the hospital-based level are still scarce, so the aim of this study was to assess the prevalence of ASD among CHD patients and its associated factors among CHD patients who used surgical and transcatheter closure intervention in Vietnam.

## 2. Methods

### 2.1. Study Setting

The study was conducted in Da Nang city, the fourth largest city in Vietnam and the largest seaside city in Central Vietnam, with a population of over 1 million people in the area of 1285 km^2^. The health system in Da Nang city is relatively developed, and it is managed and directed by the Da Nang Department of Health. At the provincial level, there are provincial hospitals and provincial preventive medicine centers. At the district level, there are district hospitals and district health centers, and, at commune level, there is commune/ward health station. As of 2019, Da Nang city has 69 health facilities, including 13 hospitals and 56 commune health stations.

Da Nang hospital is one of the largest hospitals in Central Vietnam and is responsible for treating patients in Da Nang city and neighboring provinces of South–Central Vietnam. Da Nang hospital is a general hospital with 1000 beds, which could provide treatment for all types of cardiovascular, thoracic, urological, gastrointestinal, neurological, and osteoarthritis disease. Da Nang Hospital is one of the few hospitals with open-heart surgery in Vietnam. Da Nang is the fourth largest center in Vietnam for the treatment of CHD after Hanoi, Ho Chi Minh City, and Thua Thien Hue province [17].

### 2.2. Data Sources and Data Collection

This was a cross-sectional study with the data collected from medical records of CHD patients in Da Nang hospital. The study proposal was approved by the Ethical Committee Board at Da Nang hospital in Vietnam under Decision No. 380/BVĐN-YĐ, dated 18 July 2016. The proposal was also agreed and approved by the Ethical Committee Board of the Ludwig-Maximilians-Universität München, Munich, Germany (Document LMU, project no. 18-221, dated 2 May 2018). All the personal information of the patients in the research was removed and kept confidential, only being accessed if needed by researchers. All coded data were stored and secured according to hospitals’ guidelines and requirements. Data from all patient records were collected for the period between 1 January 2010 and 31 December 2015. Inclusion criteria included all CHD patients who have been treated either by surgical or transcatheter intervention. Exclusion criteria were CHD patients who were not eligible for surgical or transcatheter intervention or those who needed only internal medication treatment. According to the inclusion and exclusion criteria, a total of 1220 CHD patients was enrolled in the study.

### 2.3. Variables

The main outcome variable in this study was the ASD status. The status of ASD was defined as whether or not a patient was diagnosed with ASD based on the physician’s diagnosis and medical record. If the patient has had surgery for ASD closure, the surgeon’s report was used to get the final diagnosis. For patients intervened by transcatheter closure, the report from the doctor who performed transcatheter closure procedure was used to get the final diagnosis. Demographic variables from patients included age, sex, and place of residence.

### 2.4. Data Collection

Members of the research team contacted Da Nang hospital to discuss specific data-collection procedures. After the plan was approved by Da Nang hospital, the research team collaborated with the relevant departments to search for the medical records. A questionnaire was developed based on the study objectives to collect data. The questionnaire was designed by experienced researchers in the research team. Because patients were not yet managed by electronic medical records, data collection was based on paper medical records. Data collection was performed by two experienced nurses with more than five years of experience in similar functions. The two nurses were trained on how to find relevant medical records and collect information for two days. Data collected by the two nurses were forwarded and revised by a doctor, who was a member of the research team, to check the logic and completeness of the data. In case of error detection or incompleteness in the data-collection process, the researcher would mark and request data collectors to fix those errors.

### 2.5. Statistical Analysis

The proportion of ASD was categorized according to the patient’s demographics, e.g., age, sex, and place of residence, using descriptive statistic methods. Quantitative data were presented as mean and standard deviation. To compare differences between two groups, Student’s *t*-test or the Mann–Whitney U test were used for quantitative variables, and Chi-square test or Fisher Exact test were used for qualitative variables. Logistic regression was used to estimate the variables influencing ASD prevalence. All statistical analyses were conducted by using STATA^®^ 14.0. The level of statistical significance was set at *p*-value less than 0.05.

## 3. Results

From January 2010 through December 2015, a total of 1220 CHD patients who used surgical and transcatheter services in Da Nang hospital were enrolled. The characteristics of the study population are shown in Table 1. The proportion of CHD patients in 2010 was the lowest (8.9%), while the proportion of CHD patients in 2011 was the highest (21.5%). The proportion of CHD patients sharply increased between 2010 and 2011, and then it gradually decreased from 2011 to 2015. The proportion of women (58.9%) was higher than that of men (41.1%). The age group of 0–9 years old constituted 70.2% of all CHD patients in this study. The majority of CHD patients came from rural areas (69.7%).

**Figure 1:**
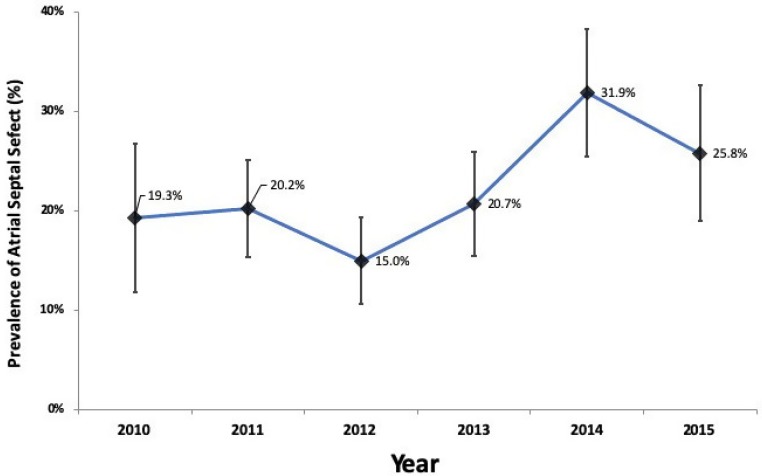
Trends in prevalence and 95% CI of atrial septal defect among congenital heart disease patients, 2010–2015.

Figure 1 presents trends in prevalence and 95% CI of ASD by year among surgical and transcatheter patient groups during the period of 2010 to 2015. The prevalence varied between time periods, ranging between 15% and 31.9%. Table 2 shows the prevalence of ASD among patients who used surgical and transcatheter closure intervention in Da Nang hospital during the period of 2010–2015. The overall prevalence of ASD among CHD patients was 18.5%. The prevalence of ASD among women (25.9%) was significantly higher than that among men (16.0%). The prevalence of ASD increased gradually from the age group of 0–9 years old to the age group of 40 years old and above. The prevalence of ASD was not different significantly between urban and rural areas.

Table 3 shows the results of multiple logistic regression analyses to explore the factors associated with ASD diagnosis. The results show that ASD occurred significantly more frequently among women participants (OR = 1.4, 95% CI = 1.02–1.9) and older age group participants (age group 10–19 years old: OR = 3.9, 95% CI: 1.9–4.3; age group 20–39 years old: OR = 6.8, 95% CI: 4.4–10.0; age group 40 years old or greater: OR = 16.0, 95% CI: 9.2–27.9).

## 4. Discussion

This was the first study conducted in Vietnam that assessed the prevalence of ASD among CHD patients who have been treated either by surgical or transcatheter-closure interventions. In this study, we found a variation in the prevalence of ASD by year from 2010 to 2015. The prevalence of ASD was lowest in 2012 and reached the highest level in 2014, and then it seemed to decline again. In addition, it was found that gender and age group were associated with the prevalence of ASD.

Globally, ASD is the second more prevalent CHD following ventricular septal defect, accounting for a total of 1.441 (1.215–1.687, 95% CI) per thousand births worldwide, corresponding to a prevalence of 15.4% of all CHD subtypes [18]. The prevalence of ASD among CHD in other Southeast Asian countries has been reported [19,20,21]. In Laos, a study was conducted between July 2013 and November 2015, evaluating 797 patients, and found a prevalence of ASD of 24.9%, from which 32% of the patients have had surgical intervention [19]. In a Thai 10-year study from 2003 to 2013, the prevalence of CHD in 27,132 adult patients who were accessed by echocardiography was 4.0%. ASD was the more prevalent type of CHD with 43%, and the ratio of women-to-men prevalence was 3.9:1 [20]. In Indonesia, from a total of 379 patients with 17 years or more who underwent congenital cardiac surgery between January 1998 and December 2006, a total of 53.8% was operated due to ASD [21]. The prevalence of ASD in our study varied between 15.0% and 31.9% in six years, so these results were in line with the prevalence found in other Southeast Asia countries [19,20,21]; however, they were slightly higher than the worldwide prevalence of ASD among CHD patients, which was estimated at 15.4% [18]. There is currently no study on genetic issues that impact the risk of CHD in Vietnam. Therefore, a future study on this issue needs to be done to better understand the fluctuations related to CHD, including ASD.

The prevalence of ASD was found to increase with increasing age, a pattern already seen in other Southeast Asian countries. In North–Central India, the distribution of prevalence of ASD in patients aged 18 years or more is 29.4% of all ages, while for children aged between 6 and 10 years, it is 11.9%, and for children aged between 11 and 17 years, it is 12.7% [22]. Another study performed in Bangladesh evaluated the effect of ASD and age on the development of hemodynamic dysfunctions, as well as the use of transcatheter closure to prevent these hemodynamic dysfunctions. The study found that, in a consecutive series of patients, individuals younger than 25 years were operated more frequently than patients older than 25 years [23]. This could suggest that most adult patients have not been appointed for surgical or transcatheter-closure interventions, which accounts for the increased prevalence in this age group. Improving in diagnosis, better access to medical technology, and the overall increase in the access to health-care systems may have contributed to the increase in the number of diagnoses at earlier disease stages and increased the success of surgical and transcatheter-closure interventions. However, the better outcomes and preserved cardiac function in younger patients is another outcome that contributes to explain the differences and why older patients are not operated so frequently [23].

The prevalence of ASD in this study was found to be no difference between urban and rural areas. Regional variations in ASD have been reported worldwide. In a study evaluating the quality of life in ASD patients, the distribution in the world was reported to be significantly different, with 22.7% being located in Asia, 58.7% in Europe, and 18.6% in North America [24]. Differences in medical practice have already been reported in the USA, with regional variations in the indication for ASD closure. After adjusting for patient characteristics, indication for ASD was more prevalent in urban hospitals and in the northeast and south regions of the USA [25]. In another study in the USA, and in spite of the consistent prevalence of ASD across regions, the closure index was driven not by constant patient pathology, but by provider-practice patterns [26]. The prevalence and incidence rates of a certain disease are also influenced by public access to the health system and the technological tools that allow diagnosis.

According to our results, some factors affecting being clinically treated for ASD either by surgical or transcatheter procedures were being a woman and older in age. Closure of ASD is recommended in all patients, even for asymptomatic patients before five years of age [2]. The main justification for early closure of ASD is the preventive approach for the development of further hemodynamic symptoms. Even if patients are asymptomatic in childhood, during adolescence and adulthood, there is a higher possibility of developing the pulmonary vascular obstructive disease, supra-ventricular arrhythmias, and becoming symptomatic [2]. Only small defects with less than 5 mm have no indication for closure, because they have a high probability of spontaneous closure. In spite of the reported safety of ASD closure techniques and high rates of survival with low morbidity rates at 5 and 10 years, age has a strong negative correlation with survival [27].

Our study found that women were more frequently diagnosed with ASD. This finding has already been reported in other regions, with female prevalence of ASD that has not been surgical or transcatheter closed being 68% and prevalence in women who underwent surgical or transcatheter closed for ASD being 65% [28]. In an epidemiological study conducted in Hungary, it was found that the number of newborns with ASD was significantly higher in women than men after adjusting for the total number of newborns, suggesting a higher incidence in women [29]. This study may explain the higher prevalence of ASD in women. Accordingly, even for women with asymptomatic ASD and well-compensated hemodynamic and cardiac function, there is an increased incidence of miscarriage, preterm delivery, and cardiac symptoms during pregnancy [30]. For these reasons, women may be more frequently appointed for ASD closure, especially before adulthood, in order to prevent complications during pregnancy. According to Vietnamese customs and habits, boys tend to be preferred over girls in the family. Therefore, men can be treated earlier and more thoroughly than women. This may also explain why the prevalence of ASD among CHD patients was higher in women than in men.

The study has some limitations that we would like to address. The study was based upon the cross-sectional design; thus, it did not allow us to conclude about the casual relationship. Although the results of the study could refer to other hospitals in the same context, the data were collected from the hospital, so this limited the possibility of extrapolation to the community. Data collection was also based on medical health records, so it might get typo errors during registry, as well as reading errors at the collection phase.

## 5. Conclusions

The prevalence of ASD among CHD patients varied over the years during the period between 2010 and 2015. Factors associated with the increased prevalence of corrected ASD were being a woman and older in age. The findings suggest that a targeted policy should be developed to provide more-specific health-care services of ASD for women and older patients.

## Figures and Tables

**Table 1:** Characteristics of congenital heart disease patients in the study, 2010–2015.

	N (%)
Year	
2010	109 (8.9)
2011	262 (21.5)
2012	254 (20.8)
2013	232 (19.0)
2014	204 (16.7)
2015	159 (13.0)
Sex	
Men	501 (41.1)
Women	719 (58.9)
Age group (year)	
0–9	856 (70.2)
10–19	167 (13.7)
20–39	125 (10.2)
40+	72 (5.9)
Area	
Urban	370 (30.3)
Rural	545 (69.7)
Total	1220 (100)

**Table 2:** Prevalence of atrial septal defect among congenital heart disease patients, 2010–2015.

	N	Atrial Septal Defect	*p* Value
		N (%)	
Sex			
Men	501	80 (16.0)	<0.01
Women	719	186 (25.9)	
Age group (year)			
0–9	856	105 (12.3)	<0.01
10–19	167	48 (28.7)	
20–39	125	62 (49.6)	
40+	72	51 (70.8)	
Area			
Urban	370	85 (23.0)	0.51
Rural	850	181 (21.3)	
Total	1220	226 (18.5)	

**Table 3:** Factors associated with atrial septal defect, 2010–2015: multivariable logistic regression analysis.

Explanatory Variable	Atrial Septal Defect	*p* Value
	OR (95% CI)	
Sex		
Men	1	
Women	1.4 (1.02–1.9)	0.04
Age group (year)		
0–9	1	
10–19	3.9 (1.9–4.3)	<0.01
20–39	6.8 (4.4–10.0)	<0.01
40+	16.0 (9.2–27.9)	<0.01
Province of residence		
Urban	1	
Rural	0.9 (0.7–1.3)	0.8
